# Novel Fractional Approach to Concrete Creep Modeling for Bridge Engineering Applications

**DOI:** 10.3390/ma18153720

**Published:** 2025-08-07

**Authors:** Krzysztof Nowak, Artur Zbiciak, Piotr Woyciechowski, Damian Cichocki, Radosław Oleszek

**Affiliations:** Faculty of Civil Engineering, Warsaw University of Technology, Al. Armii Ludowej 16, 00-637 Warsaw, Poland; artur.zbiciak@pw.edu.pl (A.Z.); piotr.woyciechowski@pw.edu.pl (P.W.); radoslaw.oleszek@pw.edu.pl (R.O.)

**Keywords:** rheology, viscoelasticity, fractal concrete creep model, prestressed concrete bridges, slag addition, air-entraining admixtures

## Abstract

The article presents research on concrete creep in bridge structures, focusing on the influence of concrete mix composition and the use of advanced rheological models with fractional-order derivatives. Laboratory tests were performed on nine mixes varying in blast furnace slag content (0%, 25%, and 75% of cement mass) and air-entrainment. The results were used to calibrate fractal rheological models—Kelvin–Voigt and Huet–Sayegh—where the viscous element was replaced with a fractal element. These models showed high agreement with experimental data and improved the accuracy of creep prediction. Comparison with Eurocode 2 revealed discrepancies up to 64%, especially for slag-free concretes used in prestressed bridge structures. The findings highlight the important role of mineral additives in reducing creep strains and the need to consider individual mix characteristics in design calculations. In the context of modern bridge construction technologies, such as balanced cantilever or incremental launching, reliable modeling of early-age creep is particularly important. The proposed modeling approach may enhance the precision of long-term structural behavior analyses and contribute to improved safety and durability of concrete infrastructure.

## 1. Introduction

Bridge structures must meet high design requirements related to durability, load-bearing capacity, and minimization of displacements (deflections). In prestressed bridge structures, one of the key factors influencing these properties is concrete creep—a phenomenon characterized by the gradual increase in strains over time under sustained loads. In bridges with large spans and complex assembly sequences, underestimating creep effects can lead to significant changes in structural geometry, increased stresses, local exceedance of material load-bearing capacity, and, in extreme cases, failure or collapse [1].

Various mechanical models are used to describe creep, including two fundamental ones: the Maxwell model (a spring and dashpot connected in series) and the Kelvin–Voigt model (a spring and dashpot connected in parallel). Over the years, numerous simplified methods for estimating creep in bridge engineering have been developed [2]. The most widely used is the effective concrete elasticity modulus method, though its accuracy is limited, particularly when accounting for load history and construction staging. For bridges built using the incremental launching method or balanced cantilever method, the influence of assembly sequence on displacements and stresses is critical and necessitates more precise analytical tools. More refined approaches to creep effects (e.g., Trost, Bažant, or incremental methods based on linear elasticity theory) rely on iterative procedures and require advanced computational implementation.

Modeling concrete creep requires consideration of both theoretical aspects and parameters derived from experimental studies. Modern models describing this phenomenon are based on empirical and mechanical relationships; however, their effectiveness in engineering practice depends on proper parameter calibration. The composition of the concrete mix—particularly the type of aggregate, as well as the type and quantity of admixtures and additives—significantly influences creep behavior. Construction chemistry is a rapidly evolving branch of the building materials industry. Contemporary concrete mixes exhibit trends such as reduced water content (lower w/c ratio) while maintaining adequate workability. To investigate the impact of mix composition on creep, material testing of industrially relevant concrete mixes is essential. Precise identification of model parameters enhances the accuracy of numerical analyses, which is crucial for designing and assessing the durability of concrete structures.

In recent decades, numerous models have been developed to predict concrete creep, reflecting both empirical observations and theoretical insights (Bažant, 2001 [3]; Neville, 1995 [4]; Flügge, 1975 [5]). Despite extensive research, accurately capturing long-term creep behavior still remains a significant challenge in structural engineering (ACI Committee 209, 1992 [6]). Traditional prediction methods, such as the effective modulus approach, are widely adopted in engineering practice due to their simplicity. However, these methods often neglect crucial aspects such as load history, material aging, and environmental effects, leading to considerable prediction inaccuracies, especially in large-scale infrastructure (Ghali et al., 2012 [7]; Hołowaty, 2013 [8]).

More advanced approaches, including the Age-Adjusted Effective Modulus method (AAEM) proposed by Bažant (1972 [9]), as well as the B3 and B4 models (Bažant and Baweja, 1995 [10]; Bažant et al., 2015 [11]), attempt to incorporate these critical factors. Yet, they typically rely heavily on empirical calibration, limiting their general applicability and predictive reliability for concretes with modified compositions or innovative admixtures (Qiang et al., 2012 [12]; Hołowaty, 2012 [13]). Given the contemporary trends toward increasingly sophisticated concrete mixtures, such as those involving high slag contents or air-entraining admixtures, there is an evident necessity for developing new modeling frameworks capable of accurately capturing the nuanced rheological responses of these materials (Buyukozturk, 2004 [14]; Gwoździewicz, 2014 [15]).

Recent literature emphasizes the benefits of fractional calculus-based rheological models, which provide significantly improved flexibility and predictive accuracy compared to classical integer-order models (Mainardi, 2010 [16]; Sapora et al., 2014 [17]). However, despite their demonstrated theoretical advantages, fractional models have not yet seen widespread integration into engineering practice, largely due to a lack of comprehensive parameter calibration procedures and practical validation (Magin, 2004 [18]). Therefore, the primary motivation behind the present study is to address these limitations, systematically calibrating fractional derivative models using experimental creep data and demonstrating their applicability to modern concrete mixes relevant to bridge engineering.

Fractional models (such as FKV and FHS) were selected due to their theoretical ability to better represent nonlinear creep behavior, concrete aging, and environmental effects compared to classical advanced models (e.g., B3, B4, AAEM). Classical models often require complex calibration or numerous empirical parameters to reflect these effects, whereas fractional models offer more flexibility with fewer parameters, thus simplifying practical applications.

By explicitly integrating recent developments and addressing critical shortcomings of existing approaches (Radomski, 2012 [1]; Nowak et al., 2025 [2]), this research contributes to filling significant gaps in the current understanding of concrete creep prediction. Moreover, it underscores the urgent need for continued research into advanced fractional models, specifically tailored to contemporary construction practices and the latest concrete technologies.

The aim of this article is to analyze the influence of concrete mix composition with particular emphasis on blast furnace slag content and air-entrainment levels on creep development, as well as to evaluate the applicability of innovative rheological models utilizing fractional-order derivatives for calibrating and predicting the long-term behavior of bridge structures. Recent studies underline the necessity of employing advanced rheological models to capture complex creep behaviors accurately, especially in modern concrete mixes modified by mineral additives and chemical admixtures. As highlighted in a comprehensive review by Ribeiro et al. (2021 [19]), fractional calculus provides powerful tools to improve prediction accuracy and address limitations of conventional creep modeling approaches. Fractional derivative models offer greater flexibility in describing the time-dependent strain characteristics of concrete, accommodating both linear and nonlinear creep phenomena observed in experimental studies.

Further supporting this perspective, Bouras and Vrcelj (2023 [20]) investigated fractional and fractal derivative-based creep models for concrete subjected to both constant and time-varying loading conditions. Their findings demonstrate that fractional-order models not only provide superior fitting to experimental creep data but also enable more robust predictions under variable loading scenarios, which are highly relevant for real-world bridge structures exposed to fluctuating traffic and environmental loads.

Additionally, Zhang et al. (2019 [21]) developed a nonlinear creep damage constitutive model for concrete based on fractional calculus theory. Their research confirmed that fractional-order models can effectively capture the nonlinear evolution of creep damage, particularly in concrete mixes incorporating supplementary cementitious materials such as blast furnace slag. This approach allows for a more accurate representation of the long-term degradation and strain processes, which is crucial for ensuring the durability and safety of bridge structures.

Therefore, this research not only contributes to validating the fractional derivative approach against experimental data but also supports its broader application in structural engineering practice. By integrating insights from recent advancements in fractional calculus-based modeling, the study aims to provide engineers with reliable tools for better long-term displacement predictions of concrete bridges. The results obtained may significantly enhance structural design methods, enabling more effective management of creep-related risks and extending the service life of critical infrastructure.

## 2. Methods for Assessing Concrete Creep in Prestressed Bridge Structures

Due to the significant share of long-term loads and the high requirements for serviceability and durability, prestressed bridge structures are among the most sensitive to rheological processes. Proper consideration of creep is particularly important in prestressed bridges constructed using the balanced cantilever method. The cyclic appearance of self-weight and prestressing during the construction of successive segments induces displacements and strains of the load-bearing structure. To achieve the designed elevation in the final configuration, so-called construction camber is used during construction, which must be precisely predicted at the design stage. During operation, the structure continues to be subjected to concrete creep, which causes long-term changes in geometry (including the designed elevation) and redistribution of internal forces and support reactions from long-term loads (self-weight and prestressing). As a result of long-term creep, span bending moments, among others, increase, leading to higher stresses and reduced crack resistance of the structure.

Over the years, numerous simplified methods for estimating the impact of creep in bridge structures have been developed [2]. The most popular is the effective concrete elasticity modulus method, in which the effect of creep is included by introducing a correction to the modulus of elasticity of concrete using the coefficient $\varphi \left(t,t_{0}\right)$ [8,22]. This method requires calculations to be performed in two independent numerical models of the structure-separately for long-term loads, accounting for the reduction in the modulus of elasticity, and for short-term loads without reduction. The results from both models must then be superimposed [22]. This approach enables the use of non-specialist FEM systems for the analysis of engineering structures. The disadvantage of this method is the inability to account for load history. In the effective concrete elasticity modulus method, creep strains are fully reversible (similarly to the Kelvin–Voigt rheological model).

Another very popular simplified method for estimating creep effects in bridge design is the $C_{creep}$ correction factor method, used to determine internal forces in uncomplicated structures built in stages, for example, spans made of precast beams. Bridge structures made of precast beams are often configured as hyperstatic systems, with reinforced concrete joints (connections) ensuring continuity. During construction, precast beams act as simply supported members. Due to creep from self-weight, the internal force system in the integrated structure tends toward the state that would exist if it were built as a continuous system from the outset. Creep from self-weight causes a slow increase in support moments. In the case of precast beams, the age of the beam at the time of installation has a significant impact on the $C_{creep}$ factor.

Advanced methods for considering concrete creep, such as the Trost method, Bažant’s method, or the incremental method based on linear elasticity theory, require an iterative approach and complex numerical implementation. For this reason, they are available only in specialized engineering software, such as SOFiSTiK, Midas, or Lusas [7,14,15]. To use these methods, it is necessary to create a precise numerical model of the structure, accounting for all intermediate stages of its behavior. This applies to both changes in the structural system (e.g., assembly and operation stages) and the history of applied loads. It is also crucial to consider the prestressing system and determine the rheological parameters of the concrete.

One of the first more accurate methods to account for creep was the so-called modified effective modulus of elasticity method (Trost method, 1967). This was a development of the classical approach based on the effective modulus, by considering the sequence of applied loads using a modified modulus of elasticity:

(1) $$∆\varepsilon_{co}\left(t_{0}\right)+∆\varepsilon_{p}\left(t,t_{0}\right)=\frac{{\Delta \sigma }_{c}\left(t_{0}\right)}{E_{co,ef}}+\frac{{\Delta \sigma }_{c}\left(t\right)-{\Delta \sigma }_{c}\left(t_{0}\right)}{E_{co,m}}$$

where

t—the age of the concrete at the considered time,

$t_{0}$—the age of the concrete at the time of permanent load application,

$∆\varepsilon_{co}\left(t_{0}\right)$—increment of elastic strain in concrete at time $t_{0}$,

$∆\varepsilon_{p}\left(t,t_{0}\right)$—increment of creep strain in concrete at time t, caused by the load applied at time $t_{0}$,

${\Delta \sigma }_{c}\left(t_{0}\right)$—increment of stress in concrete at time $t_{0}$,

$E_{co,ef}$—effective modulus of elasticity of concrete,

${\Delta \sigma }_{c}\left(t\right)$—increment of stress in concrete at time t,

$E_{co,m}$—modified modulus of elasticity of concrete, determined by the formula:

(2) $$E_{co,m}=\frac{E_{c}(t_{0})}{1+\rho (t,t_{0})\cdot \varphi \left(t,t_{0}\right)}$$

$E_{c}\left(t_{0}\right)$—modulus of elasticity of concrete at the time of load application $t_{0}$,

$\rho \left(t,t_{0}\right)$—relaxation (Trost) coefficient,

$\varphi \left(t,t_{0}\right)$—creep coefficient in the time interval from $t_{0}$ to t.

The Trost method has been implemented, among others, in the SOFiSTiK program. However, it should be noted that this approach does not account for the aging effect of creep. The inclusion of this effect is possible with the Age-Adjusted Effective Modulus method (AAEM), proposed by Bažant in 1972 [9].

In this approach, the classical modulus $E_{co,m}$ is replaced by a time-dependent value $E^{\prime \prime }\left(t,t_{0}\right)$, and the relaxation coefficient is replaced by the aging coefficient $\chi \left(t,t_{0}\right)$:

(3) $$E^{\prime \prime }\left(t,t_{0}\right)=\frac{E\left(t_{0}\right)-R(t,t_{0})}{\phi (t,t_{0})}$$

where $R(t,t_{0})$ is the relaxation function. The aging coefficient can be determined from:

(4) $$\chi \left(t,t_{0}\right)=\frac{E\left(t_{0}\right)}{E\left(t_{0}\right)-R(t,t_{0})}-\frac{1}{\phi (t,t_{0})}$$

The inclusion of aging significantly improves calculation accuracy, especially for loads applied at an early age of the concrete, which is particularly important in technologies such as balanced cantilever or incremental launching methods. The AAEM better handles loading histories that include not only relaxation but also stress increases (e.g., during long-term buckling).

In subsequent years, Bažant developed more complex models for shrinkage and creep [3]: the B3 model (Bažant and Baweja, 1995 [10]) and the B4 model. The B3 model is based on calibration with short-term measurements (1–3 months) and allows for a more accurate reflection of the properties of the designed concrete, which is especially important for high-strength concretes. Model parameters can be determined by linear regression based on short-term data. The B4 model (Bažant, Hubler, Qiang Yu [11]) introduced a modification to the calculation equation, enabling a better representation of the long-term nature of creep. This was accompanied by the development of a special database with short-term measurements, enabling calibration and verification of creep predictions.

In Bažant’s models, creep caused by a constant axial load applied at age $t_{0}$ is defined as:

(5) $$\varepsilon_{c}\left(t\right)=\sigma_{c}\left(t\right)\cdot J\left(t,t_{0}\right)$$

where the creep function has the form:

(6) $$J\left(t,t_{0}\right)=q_{1}+\;C_{0}\left(t,t_{0}\right)+\;C_{d}\left(t,t_{0},t_{c}\right)$$

where

$q_{1}$—instantaneous strain at unit stress,

$C_{0}\left(t,t_{0}\right)$—compliance function for basic creep,

$C_{d}\left(t,t_{0},t_{c}\right)$—additional compliance function for drying creep,

t—age of concrete at the time considered,

$t_{0}$—age at loading,

$t_{c}$—age at the start of drying.

The model assumes that basic creep consists of three components: aging viscoelastic, non-aging viscoelastic, and aging flow. The additional compliance function for creep accounts for drying phenomena before the first loading. Key parameters influencing the creep course include: the age of concrete at loading, concrete mix composition (cement, water, aggregate), cement type, ambient relative humidity, and effective thickness of the element.

The general incremental method is particularly useful for checking intermediate construction stages whose properties change along the structure (including structures erected using the cantilever method). The constitutive equation for concrete in the general incremental method is presented in PN-EN 1992 [23]. It accounts for both instantaneous strains caused by stress applied at time $t_{0}$, and creep caused by the long-term effect of stress applied at time t, as well as the sum of instantaneous and creep strains resulting from changes in stress in the analyzed time intervals ($t,t_{i}$), i.e., the effect of load history and shrinkage-induced strain. This allows for the inclusion of strain increments in individual calculation steps, provided the history of stress changes is known. According to the general method, the course of strains over time is analyzed incrementally, in subsequent time intervals, considering the stress value in the concrete from the previous interval. This approach allows for reflecting the dependence of creep in a given section on the stress history.

In the creep models adopted in PN-EN 1992-1-1:2008 [24], creep curves tend toward a horizontal asymptotic limit or a finite upper bound—which is not confirmed in reality, as long-term creep is logarithmic in nature, as shown in [11]. The B3 and B4 models reflect this by assuming a logarithmic course for the long-term asymptote of the creep curve. After several years, both creep and deflections increase linearly with the logarithmic scale of time.

## 3. Composition of Concrete Mixes, Laboratory Setup, and Testing Procedure

Laboratory tests were carried out as part of a doctoral dissertation [25]. The study included the preparation of nine concrete mixes differing in the mass content of blast furnace slag relative to cement and the level of air entrainment in the concrete mix. The research aimed to determine the influence of concrete mix composition on the creep coefficient.

All designed concrete mixes used Portland cement CEM I 42.5R. Granite aggregate, the most commonly used for bridge structures in Poland, was selected for the mixes. The composition of the basic concrete mix, designated as M1, was determined based on the exposure class requirements specified in PN-EN 206 [26] assuming the most demanding exposure class XF4, associated with freeze–thaw aggression, which poses a real threat to all bridge structures. According to Table F.1 in the standard [26], the maximum w/c ratio for the designed basic mix was set at 0.45, and the cement content was not less than 340 kg per 1 m^3^ of mix. However, considering the planned modifications of the composition, especially the use of a high quantity of mineral additives and the desire to achieve a concrete class not lower than C30/37, a w/c ratio of 0.4 and a minimum cement content of 450 kg/m^3^ were adopted.

The blast furnace slag content in the designed concrete mixes was assumed as follows (Table 1):

I. s/c = 0% (mixes M1, M2, M3)

II. s/c = 25% (mixes M4, M5, M6)

III. s/c = 75% (mixes M7, M8, M9)

Additionally, three levels of air entrainment were specified using the air-entraining admixture Chryso Air A10 (Table 2).

In the preliminary tests of the designed concrete mixes, the consistency and air content of the concrete mix as well as the compressive strength of the concrete were determined. In the next stage, based on the results of the preliminary tests, the compositions of the concrete mixes were prepared according to the principles of experimental design, for which the main tests were conducted.

The main tests included: compressive strength testing after 28 days of curing (cube specimens with edge d = 150 mm), modulus of elasticity testing after 28 days of curing (cylindrical specimens with diameter d = 150 mm and height h = 2d), shrinkage testing in the unloaded state over time, and total strain over time under load (including creep).

Creep testing was performed on two specimens for each mix, with an additional three specimens per mix for shrinkage measurement. The tests were conducted on prismatic specimens with dimensions d = 100 mm and L = 500 mm, prepared according to PN-EN 12350-1 [27]. Preparation, molding, and curing of the specimens were carried out in accordance with PN-EN 12390-2 [28]. The specimens were demolded after 24 h and weighed. For strain measurements, measuring studs made of anti-corrosive metal were glued to two un-troweled side surfaces of each specimen. The gauge length $L_{0}$ (distance between measuring points) was 250 mm and was located about 125 mm from both ends of the specimen. After demolding, the specimens were cured in water for the next 27 days. Following the curing period, the specimens were transferred to the creep testing room, where conditioning lasted for one day before the actual creep test commenced.

**Figure 1:**
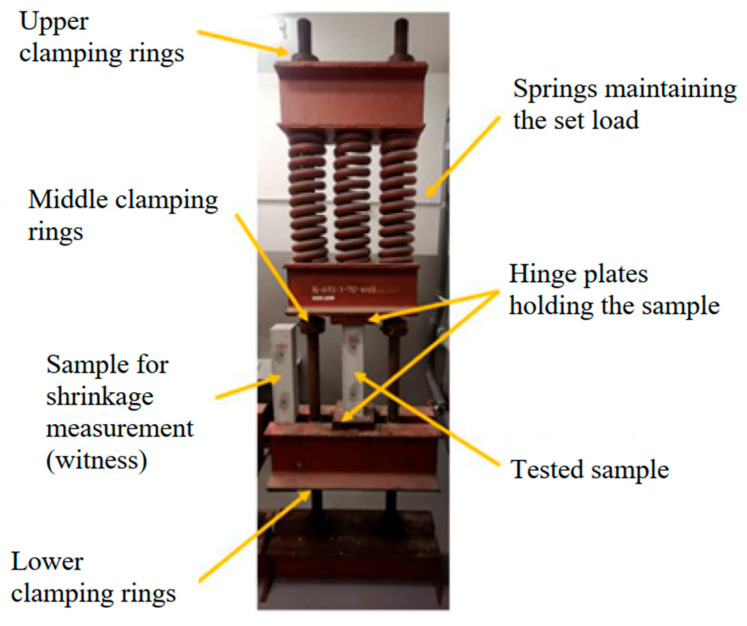
View of the creep testing apparatus (spring-type creep frame).

The creep coefficient testing was performed in accordance with PN-EN 12390-17 [29]. The basic testing apparatus, as described in the standard, consisted of loading frames called creep rigs (Figure 1).

**Figure 2:**
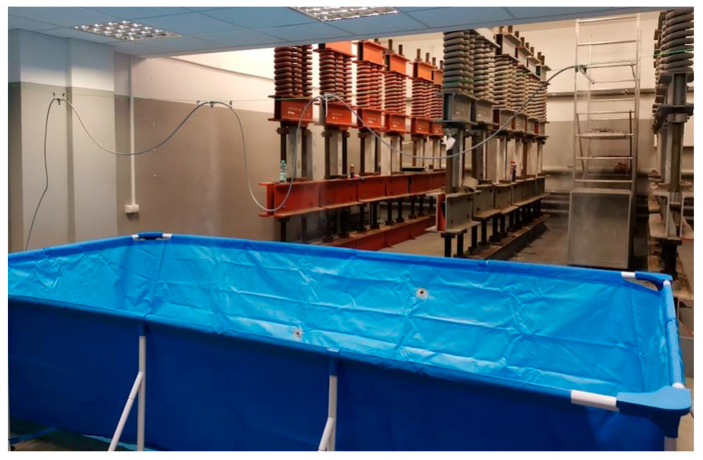
Set of spring-type creep frames used in the tests.

A set of 18 spring-type creep rigs was used for the tests (Figure 2), allowing simultaneous testing of two specimens from each mix. Each creep rig was equipped with a set of three springs, which together could transfer a load of 30 tons (approx. 300 kN).

Before placing the specimens in the creep rig, the initial gauge length $L_{0}$ was measured on both sides of each specimen. The initial and all subsequent measurements were performed using a digital micrometer with an accuracy of 0.001 mm. The specimens intended for creep testing were then placed in the creep rigs. The load was applied to the specimens using a hydraulic actuator powered by a compressor, located under the lower crossbar of the creep rig. Upon reaching the target force, the specimen position was locked by tightening the lower clamping rings, thus transferring the task of maintaining constant stress to the set of springs.

The first step involved applying a preliminary load equal to 0.25 of the target load. The gauge lengths were then measured, and the strain of the specimen was calculated on both sides. If the strains determined along the gauge lines differed by more than 20% from their mean length, the specimens were centered and the procedure was repeated until uniform loading was achieved. The target load was reached by gradually increasing the load until the test stress level of 1/3 of the compressive strength (determined in the preliminary tests) was achieved. For each specimen, immediately after applying the target load, the gauge length was measured, and the initial strain $\varepsilon_{cc}(t_{0})$ was calculated as the mean value for the mix type. The devices maintained the set load with an accuracy of ±3% throughout the test. During the test, the laboratory temperature was kept at 20 °C ± 2% and humidity at 80% ± 5%. Subsequent measurements were taken daily for one week, then weekly for one month, and finally monthly until the last measurement on day 365 after loading. For all these measurements, the strains $\varepsilon_{cc}(t)$ were calculated. In parallel, shrinkage measurements were performed on control specimens, from which the shrinkage strain $\varepsilon_{cs}(t,t_{0})$ was calculated. The creep strain at time, t, was determined using the formula:

(7) $$\varepsilon_{cc}(t,t_{0})=\varepsilon_{cc}(t)-\left[\varepsilon_{cs}\left(t,t_{0}\right)+\varepsilon_{cc}\left(t_{0}\right)\right]$$

where

$\varepsilon_{cc}(t)$—total strain of loaded specimens at time t,

$\varepsilon_{cs}(t,t_{0})$—shrinkage strain of control specimens at age t, determined according to PN-EN 12390-16 [30];

$\varepsilon_{cc}(t_{0})$—initial strain after application of load.

Based on the creep strain, the value of the creep coefficient was determined using formula:

(8) $$\varphi (t,t_{0})=\varepsilon_{cc}(t,t_{0})\cdot \frac{E_{c}}{\sigma_{c}\left(t_{0}\right)}$$

where

$\varepsilon_{cc}(t,t_{0})$—creep strain,

$E_{c}$—secant modulus of elasticity,

$\sigma_{c}(t_{0})$—constant applied stress at time $t_{0}$.

(9) $$E_{c}={1.05\cdot E}_{cm}$$

$E_{cm}$—the modulus of elasticity after 28 days of curing.

## 4. Laboratory Test Results

The results of the compressive strength tests after 28 days of curing and the modulus of elasticity tests are presented in Table 3 and Table 4, respectively.

The values of total, creep, and shrinkage strains were calculated based on measurements of loaded concrete specimens (shrinkage + creep) and control specimens (shrinkage only). The measurement results were averaged for concretes made from mixes M1–M9 and then, by referencing the initial measurements, recalculated as strains for each day of the testing procedure.

The values of the creep coefficients for t = 365 days were determined using Formula (8) and are presented in Table 5.

After completing the tests described in Section 3, the specimens continued to be subjected to the target load. For the purpose of more accurate calibration of the rheological model parameters, additional measurements were taken on days 405, 579, and 1316 after the application of the load.

**Figure 3:**
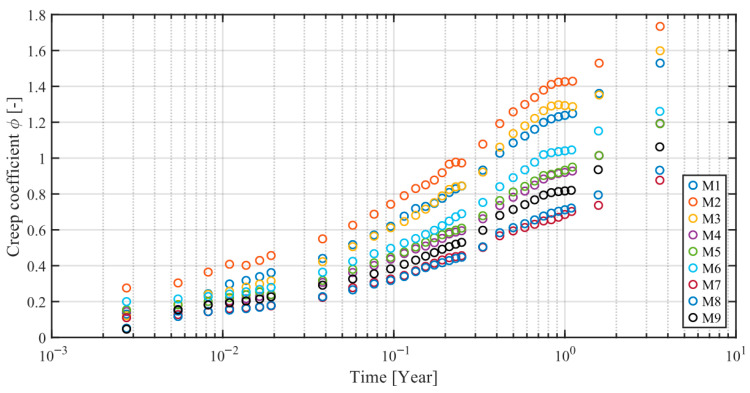
Comparison of creep coefficient curves obtained for all tested concrete mixes (M1–M9).

For improved clarity, Figure 3 presents a comparison of creep coefficient curves obtained for all tested concrete mixes (M1–M9).

## 5. Mechanical Models of Concrete Creep

Concrete creep is the time-dependent change in strain occurring in concrete subjected to prolonged stress. It significantly impacts structural behavior, durability, and long-term stability, particularly in infrastructure like prestressed concrete bridges. Accurately modeling concrete creep is crucial for predicting structural serviceability, deflection, and stress redistribution, thereby preventing potential failures.

Concrete creep modeling encompasses diverse approaches aimed at accurately capturing the material’s complex, time-dependent behavior [31]. Traditional methods often rely on empirical formulations based on long-term experimental observations [4]. Another approach utilizes phenomenological models that characterize creep using mathematical equations directly fitted to experimental data [6]. Mechanical rheological models, such as Maxwell and Kelvin–Voigt, represent another influential category, explicitly modeling fundamental material phenomena through idealized mechanical components [5]. These mechanical models combine elastic springs, representing instantaneous strain and energy storage, and viscous dashpots, modeling time-dependent strain and energy dissipation [32]. More advanced methods involve fractional calculus, enhancing traditional mechanical models by employing fractional derivatives to more precisely simulate viscoelastic behavior [16].

### 5.1. Classical Mechanical Models

Classical mechanical models describe the rheological behavior of concrete by combinations of linear elastic springs representing instantaneous strain and viscous dashpots representing time-dependent strain. The most commonly referenced classical models are the Maxwell model and the Kelvin–Voigt model.

**Figure 4:**
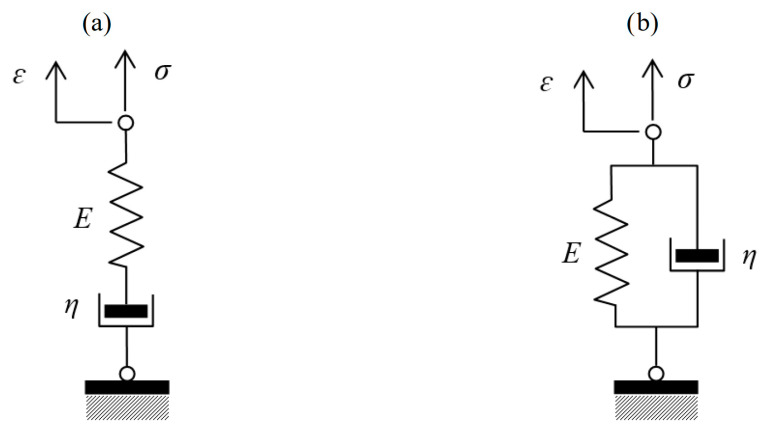
Classical rheological models: (**a**) Maxwell and (**b**) Kelvin–Voigt.

The Maxwell model (Figure 4a), composed of a spring and a dashpot arranged in series, effectively captures stress relaxation but is unsuitable for accurately modeling creep under sustained loading, as it predicts a linear, unbounded increase in strain over time, ultimately approaching infinity. Its differential equation is as follows

(10) $$\dot{\varepsilon }(t)=\frac{1}{E}\dot{\sigma }(t)\cdot \frac{\sigma \left(t\right)}{\eta }$$

where the superimposed dot denotes differentiation with respect to the time coordinate, thus $\dot{\varepsilon }\equiv d\varepsilon /dt$ and $\dot{\sigma }\equiv d\sigma /dt$. Moreover $\sigma \left(t\right)$ represents stress time function, ε(t) is strain function, E [Pa] modulus of elasticity, and η [Pa·s] viscosity coefficient.

The Kelvin–Voigt model (Figure 4b), with parallel spring and dashpot elements, effectively represents creep behavior under constant stress, characterized by immediate elastic strain and delayed viscous flow. Its differential equation is

(11) $$\eta \;\dot{\varepsilon }\left(t\right)+E\;\varepsilon (t)=\sigma \left(t\right)$$

For constant stress $\sigma_{0}=const$, the solution of the equation describing creep is

(12) $$\varepsilon (t)=\frac{\sigma_{0}}{E}\left[1-exp\left(-\frac{t}{\lambda }\right)\right]$$

where λ=η/E denotes the retardation time, indicative of the material’s delayed response.

It is worth mentioning that the Maxwell and Kelvin–Voigt models can be generalized by adding more branches. Using more branches helps to better fit experimental data. However, this approach also means that many additional parameters need to be determined. The problem of determining many parameters can be avoided by using fractional-order models. This approach is discussed in the following section.

### 5.2. Fractional Derivatives in Rheological Modeling

Fractional calculus introduces derivatives and integrals of non-integer (fractional) order, enabling improved modeling capabilities for complex viscoelastic materials like concrete [18,33]. Fractional derivatives, typically defined using Riemann–Liouville or Caputo definitions [34,35], bridge elastic and viscous behavior, offering more accurate and versatile modeling than integer-order differential equations [16,35,36].

The Riemann–Liouville fractional derivative of order $\alpha \in \left(0,1\right)$ is defined as:

(13) $$D^{\alpha }\;f\left(t\right)=\frac{f\left(0\right)}{\Gamma \left(1-\alpha \right)t^{\alpha }}+\frac{1}{\Gamma \left(1-\alpha \right)}\int_{0}^{t}\frac{\dot{f}(\xi )}{{\left(t-\xi \right)}^{\alpha }}d\xi$$

where $\Gamma \left(x\right)$ denotes the gamma function, which can be defined in two ways

(14) $$\Gamma \left(x\right)=\left\{\begin{array}{ll} \int_{0}^{\infty }t^{x-1}\exp\left(-t\right)dt & for\;x>0\\ \underset{n\to \infty }{\lim}\frac{n!\;n^{x-1}}{x\left(x+1\right)\left(x+2\right)\cdots \left(x+n-1\right)} & for\;any\;x \end{array}\right.$$

**Figure 5:**
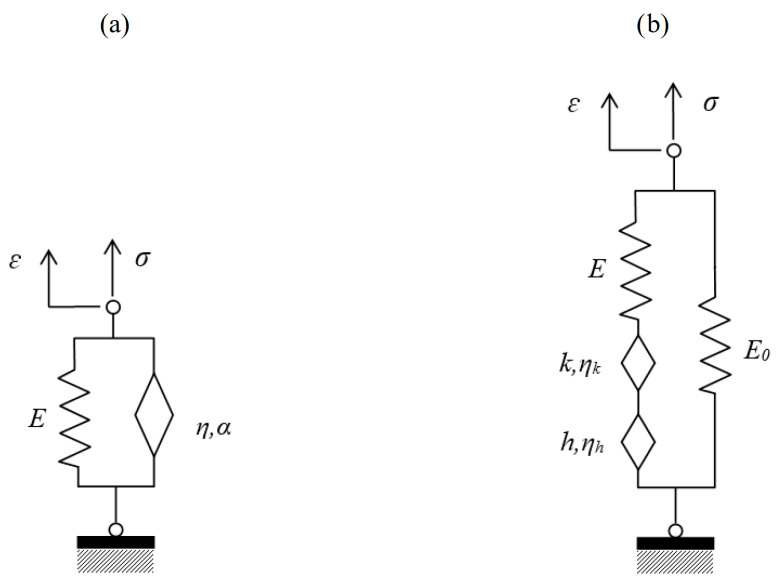
Fractional rheological models: (**a**) Fractional Kelvin–Voigt and (**b**) Fractional Huet–Sayegh.

The Fractional Kelvin–Voigt (FKV) model enhances the classical Kelvin–Voigt model by replacing the viscous dashpot with a fractional derivative element (see Figure 5a). It combines an elastic spring and a fractional-order viscous element, defined by the following fractional differential equation

(15) $$\eta \;D^{\alpha }\varepsilon \left(t\right)+E\varepsilon \left(t\right)=\sigma \left(t\right)$$

Under sustained stress $\sigma_{0}=const$, the creep strain solution is expressed using the Mittag-Leffler function $E_{\alpha ,\beta }\left(z\right)$

(16) $$\varepsilon \left(t\right)=\frac{\sigma_{0}}{E}{\left(\frac{t}{\tau }\right)}^{\alpha }E_{\alpha ,\alpha +1}\left[-{\left(\frac{t}{\tau }\right)}^{\alpha }\right],\;\;\;\tau ={\left(\frac{\eta }{E}\right)}^{1/\alpha },\;\;\;E_{\alpha ,\beta }\left(z\right)=\sum_{k=0}^{\infty }\frac{z^{k}}{\Gamma (\alpha k+\beta )}$$

The Mittag-Leffler function generalizes the exponential function, providing solutions to fractional-order differential equations. The creep compliance function $J\left(t\right)$ derived from this model can be obtained by assuming $\sigma_{0}=1\;Pa$ in Equation (16). Thus, we obtain

(17) $$J\left(t\right)=\frac{1}{E}{\left(\frac{t}{\tau }\right)}^{\alpha }E_{\alpha ,\alpha +1}\left[-{\left(\frac{t}{\tau }\right)}^{\alpha }\right]$$

Compared to classical models, the fractional Kelvin–Voigt model significantly improves fitting accuracy with experimental creep data, particularly under varying environmental conditions.

The fractional Huet–Sayegh model (FHS) further generalizes rheological modeling by combining two elastic springs with two fractional-order viscous elements, each characterized by unique retardation times (see Figure 5b). It is governed by more complex equations involving multiple fractional derivatives, allowing an extremely precise representation of creep behaviors, including nonlinear and environmental-sensitive scenarios [17].

The governing fractional-order differential equation of the FHS model can be derived by applying the stress equilibrium equation as well as the total strain rate equation in non-elastic branch. It leads to the following formula

(18) $$\dot{\sigma }\left(t\right)+{\tau_{k}}^{-k}D^{1-k}\sigma \left(t\right)+{\tau_{h}}^{-h}D^{1-h}\sigma \left(t\right)=\left(E+E_{0}\right)\dot{\varepsilon }\left(t\right)+\cdots \;{+E}_{0}\left[{\tau_{k}}^{-k}D^{1-k}\varepsilon \left(t\right)+{\tau_{h}}^{-h}D^{1-h}\varepsilon \left(t\right)\right]$$

where

(19) $$\tau_{k}={\left(\frac{\eta_{k}}{E}\right)}^{1/k},\;\;\;\tau_{h}={\left(\frac{\eta_{h}}{E}\right)}^{1/h}$$

Equation (18) describes the relationship between stress and strain for the Huet–Sayegh model in the time domain. It includes fractional-order derivatives, reflecting complex viscoelastic behavior with memory effects. These fractional derivatives complicate solving the equation analytically because conventional solution techniques developed for integer-order differential equations generally do not apply. Consequently, explicit analytical solutions are difficult to obtain, and numerical methods are typically necessary.

Due to the difficulties in solving Equation (18) directly in the time domain and expressing the creep response explicitly using the Mittag-Leffler function, we propose an alternative approach based on the Laplace transform. Applying the Laplace transform to both sides of Equation (18) yields the following equation

(20) $$\bar{\sigma }\left(s\right)=\bar{E}\left(s\right)\bar{\varepsilon }(s)$$

where $\bar{\sigma }\left(s\right)=L\left\{\sigma (t)\right\}$ and $\bar{\varepsilon }\left(s\right)=L\left\{\varepsilon (t)\right\}$ are the Laplace transforms of the stress and strain states, respectively. Moreover, $\bar{E}\left(s\right)$ denotes the so-called transfer function resulting from Equation (18) and being as follows

(21) $$\bar{E}\left(s\right)=E_{0}+\frac{E}{1+{\left(s\;\tau_{k}\right)}^{-k}+{\left(s\;\tau_{h}\right)}^{-h}}$$

The creep response of the Huet–Sayegh model can be obtained by applying an inverse Laplace transform procedure, as described in [37]. Since the Huet–Sayegh model itself already includes fractional-order elements, obtaining an explicit analytical expression for the inverse Laplace transform can be challenging, especially when fractional parameters have complex or general values. In such cases, the inversion process cannot be easily carried out analytically, and numerical methods must be employed instead. These numerical algorithms, described in detail in the literature [38,39], typically involve numerical contour integration or other approximation techniques, providing accurate results even when analytical forms are not available.

Using fractional derivatives can better account for complex, long-term concrete behaviors often overlooked in classical rheological models. The choice of fractional order significantly affects how precisely the creep curves fit real-world measurements, making its proper selection critical. Fractional calculus enables modeling of concrete’s gradual stiffness reduction during extended load application more accurately. By adjusting fractional parameters, ones can easily adapt models to varying environmental conditions like humidity or temperature. Detailed sensitivity analyses of fractional derivative parameters could highlight their individual impact on long-term creep predictions. Advanced fractional models, when combined with modern numerical algorithms, allow engineers to optimize structural designs effectively. Incorporating fractional derivatives into existing software for structural analysis could greatly enhance predictions of time-dependent strains. Compared to classical advanced models (e.g., B3, B4), fractional derivative models more naturally accommodate nonlinear creep and aging effects with simpler parameter calibration.

## 6. Parameter Calibration of Fractional Models

Calibrating fractional models to experimental data involves determining optimal values of parameters such as E, η, and α in case of the FKV model (see Figure 5a). Calibration typically employs least-squares methods, minimizing the error between predicted and measured creep strains. Specialized software tools, often implemented in computational environments like MATLAB R2024b, facilitate the calibration process, incorporating advanced numerical algorithms.

Accurately calibrated fractional models can reliably predict concrete creep behavior for extended service life durations, commonly up to 100 years or beyond, essential for infrastructure safety and durability.

### 6.1. Curve-Fitting Procedure

The calibration of fractional rheological models, such as fractional Kelvin–Voigt (FKV) and fractional Huet–Sayegh (FHS), involves solving an optimization problem aimed at minimizing the discrepancy between experimental data and model predictions. This optimization task is mathematically expressed using a least-squares curve-fitting approach. Assuming experimentally measured creep strains at discrete time points $t_{i}$ are denoted by $\varepsilon_{exp}\left(t_{i}\right)$, and strains predicted by the fractional model with parameters $p\in R^{m}$ by $\varepsilon_{mod}(t_{i})$, the optimization problem can be defined as finding the optimal set of parameters $p_{opt}\in R^{m}$ that minimizes the following objective function

(22) $$p_{opt}=\arg\underset{p\in \Omega }{\min}{\sum_{i}\left[\varepsilon_{exp}\left(t_{i}\right)-\varepsilon_{mod}(t_{i},p)\right]}^{2}$$

where Ω is the admissible parameter space, bounded by lower parameter limits $p_{L}\in R^{m}$ and upper parameter limits $p_{U}\in R^{m}$:

(23) $$\Omega =\left\{p\in R^{m}:\;\;\;p_{L}\leq p\leq p_{U}\right\}$$

where m denotes the number of fitting parameters (3 for FKV and 6 for FHS).

In practice, solving this optimization task typically involves numerical algorithms, such as the *lsqcurvefit* function available in MATLAB R2024b, ensuring efficient convergence to the best-fitting model parameters. The solver iteratively adjusts parameter values until convergence criteria are met, optimizing the fit between experimental creep data and the model’s predictions.

### 6.2. Curve-Fitting Results

The Fractional Kelvin–Voigt model was used to describe concrete creep. This model includes three parameters requiring calibration (see Figure 5a): E [Pa], η [Pa·s^α^], and α [1]. Instead of η the parameter τ [s] can be used as it was presented in Equation (16).

Fitting the parameters of the rheological model to the experimental results was performed using a custom calibration spreadsheet developed in the MATLAB R2024b environment. The spreadsheet imported data including the measurement times and creep coefficients determined from the specimen strains. The algorithm implemented the equation of the creep coefficient Function (17), specified initial parameter values, and their permissible ranges. Calibration was performed using the least-squares method, utilizing the *lsqcurvefit* function, which enabled optimal fitting of the function parameters to the test results.

After calibrating the model, it is possible to predict the value of the creep coefficient for any time horizon, e.g., after 100 years, which corresponds to the typical design life of bridge structures.

The calibration quality of the fractional Kelvin–Voigt model was evaluated using standard statistical metrics. The average root mean square error (RMSE) for all mixes (M1–M9) was approximately 0.06, and the coefficient of determination (R^2^) values ranged from 0.92 to 0.96, indicating good agreement with experimental data. The obtained RMSE and R^2^ values are consistent with typical calibration quality reported in recent studies on fractional creep models, see e.g., [19,20,21]. A brief sensitivity analysis confirmed that the fractional order parameter (α) has the strongest impact on creep predictions, followed by viscosity (η), and modulus of elasticity (E).

**Figure 6:**
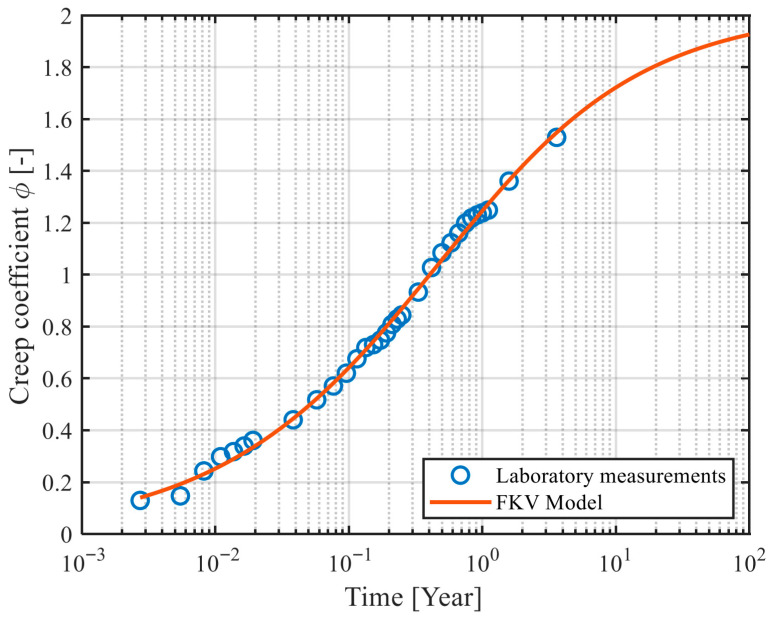
Creep coefficient function plot obtained after calibration to laboratory results of M1 samples.

**Figure 7:**
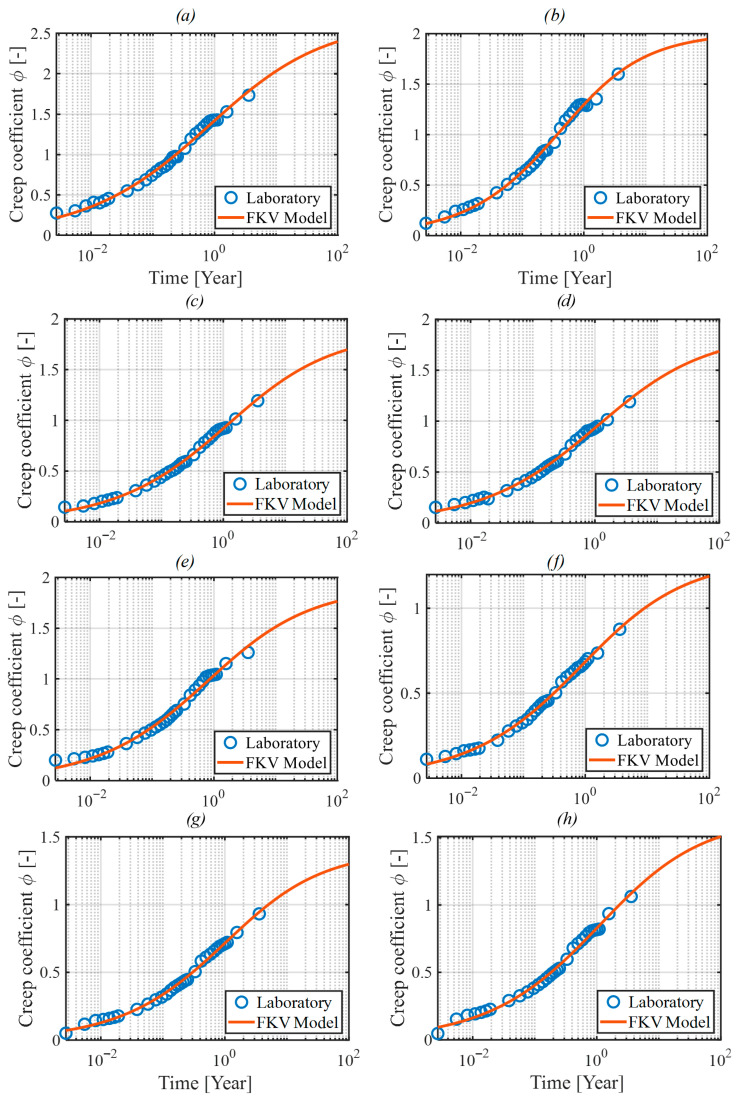
Creep coefficient function plots obtained after calibration to laboratory results of samples: (**a**) M2, (**b**) M3, (**c**) M4, (**d**) M5, (**e**) M6, (**f**) M7, (**g**) M8, (**h**) M9.

Figure 6 and Figure 7, respectively, show the plots of the creep coefficient function after calibration for samples M1–M9, including additional measurements taken at 405, 579, and 1316 days. Table 6 summarizes the values of the obtained function parameters, while Table 7 presents the values of the creep coefficient after 100 years, determined both on the basis of the calibrated functions and according to the Eurocode methodology [23,24].

**Figure 8:**
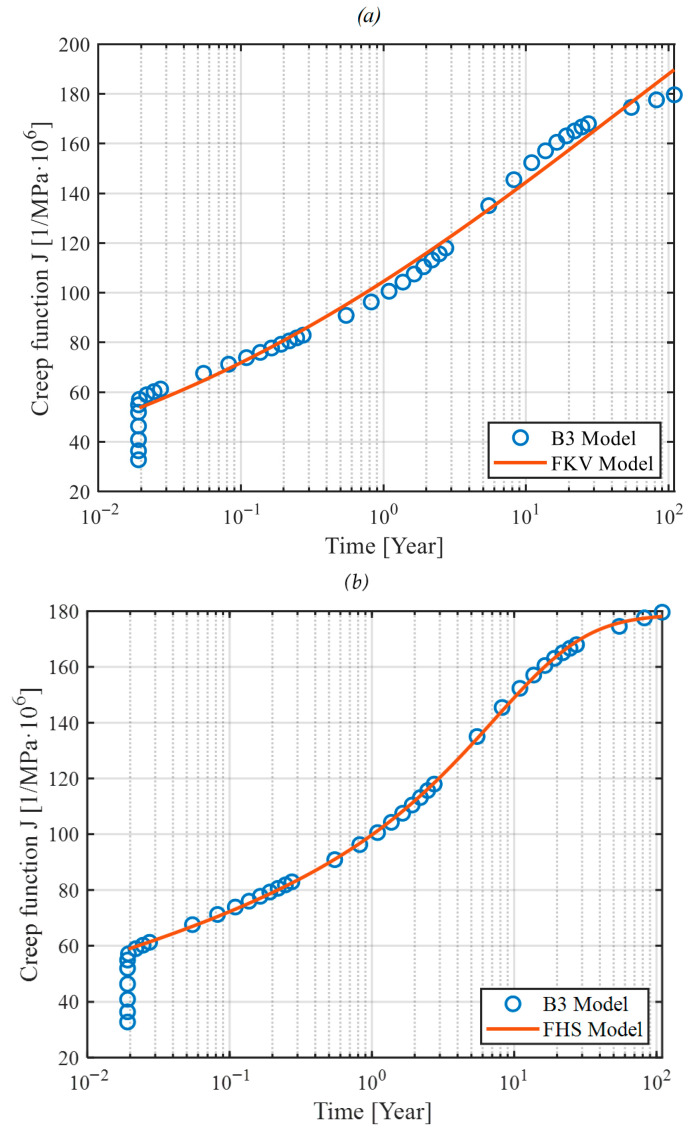
Example of model calibration to experimental data for 40% humidity: (**a**) fractal Kelvin–Voigt model, (**b**) fractal Huet–Sayegh model.

Fractional derivative models, including fractional Kelvin–Voigt and Huet–Sayegh, have demonstrated superior performance in experimental validations. Their ability to fit observed data accurately, particularly under challenging conditions such as varying humidity or temperature, highlights their practicality for engineering applications. For typical environmental conditions (relative humidity 50–80%), the fractional Kelvin–Voigt model offers excellent predictive capabilities. However, under extreme environmental conditions (e.g., humidity < 50%), the fractional Huet–Sayegh model becomes necessary due to its increased complexity and modeling flexibility (Figure 8).

It should be noted that our recommendation regarding model selection is based primarily on general theoretical considerations and practical engineering experience. The FKV model is recommended for typical environmental conditions (humidity range 50 to 80%), while the FHS model is suggested for extreme conditions (humidity below 50%). Clear quantitative criteria defining exact limits of applicability for each model require additional systematic studies. Determining precise quantitative boundaries for humidity or other environmental factors will be a subject of future research.

While this study primarily focused on gathering comprehensive macroscopic experimental data and the initial calibration of fractional rheological models, further in-depth discussions of the continuation model remain an important next step. Future studies should address the statistical robustness of the continuation model, including sensitivity analysis and validation against independently collected data. Such analysis would provide valuable insights into the long-term predictive capabilities of fractional derivative models and their practical application limitations.

Due to the high complexity and time-consuming nature of our experimental campaign, calibration of the fractional models was conducted solely using our own comprehensive dataset. However, it should be emphasized that our fractional model captures all significant phenomena included in sophisticated classical models such as B3 or B4 (e.g., nonlinear creep, aging effects, and environmental sensitivity). Additionally, the fractional model offers clear advantages including fewer parameters to fit, ease of calibration, and a well-defined mechanical interpretation based on fractional-order differential equations. Such calibrated models can easily be adapted to various loading scenarios, not limited exclusively to creep. These benefits suggest broader applicability in structural engineering practice.

This research focused primarily on evaluating whether slag content and air entrainment significantly influence concrete creep, rather than explaining the exact microstructural mechanisms behind these effects. Due to the complexity and broad scope of microstructural changes caused by slag and admixtures, a detailed interpretation (e.g., SEM, porosity, hydration studies) was beyond the scope of this paper. The microstructural origins of creep changes observed in concretes modified by slag and air entrainment will be systematically investigated in future research.

## 7. Conclusions

This research clearly showed how blast furnace slag affects concrete creep [25]. Higher slag content in concrete reduced the creep coefficient. Adding 25% slag decreased creep by about 17%. Further increasing slag to 75% resulted in an additional reduction of about 23%. However, high slag content can slow early concrete strength gain. Fast strength gain is essential for bridges built using prestressed methods such as incremental launching or balanced cantilever.

The study highlighted problems with the current Eurocode 2 procedures. Eurocode methods do not accurately predict creep for modern concrete mixes containing additives or admixtures. Differences between Eurocode predictions and laboratory results ranged from −1% to 64%. The largest discrepancy (64%) appeared in slag-free concrete designed for prestressed bridges.

The main novelties of this research are:

- A new method using fractional calculus to predict concrete creep. Fractional Kelvin–Voigt and Fractional Huet–Sayegh models provided better results compared to standard methods, accurately describing the long-term logarithmic behavior of creep.
- Clear experimental evidence showing how slag content and air entrainment affect creep, helping engineers better design concrete mixes.
- Demonstrating significant inaccuracies (up to 64%) in creep predictions by Eurocode methods, proving the need for better models.
- Emphasizing the importance of future studies on concrete creep behavior under early-age loading (around 3 days after concreting), relevant to actual bridge construction methods.

Future studies should further explore fractional models, simplify parameter selection, and link fractional-order parameters with concrete microstructure. Practical guidelines for engineers would encourage wider use of fractional models, improving the durability and safety of concrete bridges.

## Figures and Tables

**Table 1:** Composition of Concrete Mixes.

**I. Concrete mix composition with blast furnace slag content s/c = 0%**				
Cement [kg]	Slag [kg]	Water [kg]	Sand [kg]	Aggregate [kg]
466.0	0.0	186.4	616.0	1143.0
**II. Concrete mix composition with blast furnace slag content s/c = 25%**				
Cement [kg]	Slag [kg]	Water [kg]	Sand [kg]	Aggregate [kg]
405.2	101.3	186.4	601.4	1115.9
**III. Concrete mix composition with blast furnace slag content s/c = 75%**				
Cement [kg]	Slag [kg]	Water [kg]	Sand [kg]	Aggregate [kg]
321.4	241.0	186.4	581.8	1079.4

**Table 2:** Levels of Air Entrainment in Concrete Mixes.

Mix Composition Characteristics		Air Entrainment of the Concrete Mix (Chryso Air A10 Admixture)		
		1.0–3.9 [%]	4.0–5.9 [%]	6.0–7.9 [%]
s/c [%]	0	M1	M2	M3
		admixture = 0.0 [% mc]	admixture = 0.1 [% mc]	admixture = 0.2 [% mc]
		slump = 2.0 cm	slump = 3.5 cm	slump = 2.5 cm
		air content = 2.7 [%]	air content = 4.6 [%]	air content = 7.1 [%]
	25	M4	M5	M6
		admixture = 0.0 [% mc]	admixture = 0.1 [% mc]	admixture = 0.2 [% mc]
		slump = 1.8 cm	slump = 3.8 cm	slump = 2.5 cm
		air content = 2.4 [%]	air content = 4.0 [%]	air content = 7.0 [%]
	75	M7	M8	M9
		admixture = 0.0 [% mc]	admixture = 0.1 [% mc]	admixture = 0.2 [% mc]
		slump = 1.5 cm	slump = 1.9 cm	slump = 1.5 cm
		air content = 2.3 [%]	air content = 4.5 [%]	air content = 6.6 [%]

**Table 3:** Compressive strength of concrete made from mixtures M1–M9 measured after 28 days of curing (typical variability approx. ±2–6%).

Mix Composition Characteristics		Concrete Compressive Strength After 28 Days of Curing [MPa]		
		Air Content of the Mixture		
		Admixture = 0.0 [% mc]	Admixture = 0.1 [% mc]	Admixture = 0.2 [% mc]
s/c [%]	0	M1	M2	M3
		f_cm_ = 54.99	f_cm_ = 51.13	f_cm_ = 46.34
	25	M4	M5	M6
		f_cm_ = 60.93	f_cm_ = 57.36	f_cm_ = 51.04
	75	M7	M8	M9
		f_cm_ = 65.62	f_cm_ = 57.48	f_cm_ = 52.17

**Table 4:** Elastic modulus of concrete measured after 28 days of curing for mixtures M1–M9 (typical variability approx. ±2–4%).

Mix Composition Characteristics		Concrete Compressive Strength after 28 Days of Curing [MPa]		
		Air Content of the Mixture		
		Admixture = 0.0 [% mc]	Admixture = 0.1 [% mc]	Admixture = 0.2 [% mc]
s/c [%]	0	M1	M2	M3
		E_cm_ = 31,350	E_cm_ = 30,800	E_cm_ = 30,000
	25	M4	M5	M6
		E_cm_ = 33,450	E_cm_ = 32,000	E_cm_ = 30,000
	75	M7	M8	M9
		E_cm_ = 34,900	E_cm_ = 32,300	E_cm_ = 30,500

**Table 5:** Strain and creep coefficient values according to the laboratory procedure for t = 365 days after loading (typical variability approx. ±5–10%).

Sample Designation	Strain				Creep Coefficient
	Instantaneous	Total	Shrinkage	Creep	
	$\varepsilon_{cc}(t_{0})$	$\varepsilon_{cc}(t=365d)$	$\varepsilon_{cs}(t=365d)$	$\varepsilon_{cc}(t=365d,t_{0})$	$\varphi (t=365d,t_{0})$
	(1)	(2)	(3)	(4) = (2) − ((3) + (1))	(5) = (4)/(1)
M1	0.00051	0.00150	0.00036	0.00063	1.238
M2	0.00050	0.00155	0.00034	0.00071	1.426
M3	0.00046	0.00144	0.00038	0.00060	1.293
M4	0.00058	0.00147	0.00035	0.00054	0.920
M5	0.00062	0.00154	0.00035	0.00058	0.933
M6	0.00058	0.00154	0.00037	0.00060	1.040
M7	0.00060	0.00130	0.00029	0.00041	0.685
M8	0.00059	0.00129	0.00028	0.00042	0.712
M9	0.00050	0.00116	0.00027	0.00040	0.817

**Table 6:** Optimal parameter values of the creep coefficient function using FKV model.

Sample Designation	E[GPa]	$\eta [GPa\cdot s^{\alpha }]$	α[1]	τ[Year]
M1	492.9	420.1	0.4909	0.7224
M2	374.4	417.4	0.4152	1.299
M3	496.9	388.9	0.5360	0.6332
M4	532.0	704.0	0.4536	1.854
M5	534.2	682.0	0.4431	1.736
M6	521.5	577.1	0.4583	1.247
M7	770.9	888.0	0.4571	1.363
M8	709.9	890.7	0.4815	1.602
M9	602.8	773.7	0.4591	1.722

**Table 7:** Creep coefficient values after 100 years determined using functions calibrated to laboratory results and values calculated according to Eurocode.

Sample Designation	$\varphi_{LAB}(t=100y,t_{0}=28d)$ [−]	$\varphi_{EC}(t=100y,t_{0}=28d)$ [−]	$\frac{\varphi_{LAB}-\varphi_{EC}}{\varphi_{EC}}$
M1	1.926	1.368	41%
M2	2.398	1.461	64%
M3	1.942	1.598	22%
M4	1.697	1.249	36%
M5	1.686	1.318	28%
M6	1.765	1.464	21%
M7	1.189	1.169	2%
M8	1.298	1.315	−1%
M9	1.507	1.435	5%

## Data Availability

The original contributions presented in this study are included in the article. Further inquiries can be directed to the corresponding author.

## Abbreviations

The following abbreviations are used in this manuscript:

AAEM	Age-Adjusted Effective Modulus method
FEM	Finite Element Method
FHS	Fractional Huet–Sayegh model
FVK	Fractional Kelvin–Voigt model
